# Engaging with Community Researchers for Exposure Science: Lessons Learned from a Pesticide Biomonitoring Study

**DOI:** 10.1371/journal.pone.0136347

**Published:** 2015-08-26

**Authors:** Paul Teedon, Karen S. Galea, Laura MacCalman, Kate Jones, John Cocker, John W. Cherrie, Martie van Tongeren

**Affiliations:** 1 School of Engineering and the Built Environment, Glasgow Caledonian University, Glasgow, United Kingdom; 2 Centre for Human Exposure Science, Institute of Occupational Medicine (IOM), Riccarton, Edinburgh, United Kingdom; 3 Health and Safety Laboratory (HSL), Buxton, United Kingdom; 4 School of Life Sciences, Heriot Watt University, Edinburgh, United Kingdom; Ghent University, BELGIUM

## Abstract

A major challenge in biomonitoring studies with members of the general public is ensuring their continued involvement throughout the necessary length of the research. The paper presents evidence on the use of community researchers, recruited from local study areas, as a mechanism for ensuring effective recruitment and retention of farmer and resident participants for a pesticides biomonitoring study. The evidence presented suggests that community researchers’ abilities to build and sustain trusting relationships with participants enhanced the rigour of the study as a result of their on-the-ground responsiveness and flexibility resulting in data collection beyond targets expected.

## Introduction

The use of pesticides is a subject that gives rise to public concern. The Royal Commission on Environmental Pollution (RCEP) published a report on bystander and resident exposure to pesticides and recognised that the epidemiological literature showing associations of chronic fatigue and multiple chemical sensitivity with pesticide exposure was plausible but equivocal [1]. This report and the responses to it have ensured that the issue has remained in the public eye [2–4]. Our overall study aimed to assess exposure to pesticides for adults and children living adjacent to agricultural land and investigate if exposures were elevated following relevant spray events. In addition, it sought to gauge whether the methods for assessing potential exposures in nearby residents that are used as part of regulatory risk assessment for agricultural pesticides in the UK are sufficiently conservative. To achieve these aims, we needed to recruit farmers who sprayed their crops with selected pesticides (captan, chlorpyrifos, chlormequat, cypermethrin, and penconazole) to provide their spray-event information, and to collect urine samples and activity data from residents (adults and children) living within 100m of these fields during, and for, a limited period, outwith the spray season.

We had to overcome a number of challenges. The relatively short biological half-life of modern pesticide compounds and their metabolites in the human body presents a major challenge to linking biological monitoring data to specific spray events. Farming activities involving the application of pesticides are inherently unpredictable because of changes in the weather and in the presence of insects or other potentially damaging infestations. It is good practice for farmers to spray under dry and calm weather conditions.[5] In addition, the type of crop infestation will determine the most appropriate pesticide. Whilst good practice, there is no legal obligation for farmers to inform residents of their pesticide spray activities [5], which clearly has implications for attempts at linking spray events with biomonitoring activities.

These methodological and practical issues are compounded by difficulties in the recruitment and retention of participants in biomonitoring studies. This is reflected in the wider, often health-related literature, where attrition of participants can impact on study validity [6]. Ribisl et al [7] note particular impacts on longitudinal (panel) studies and this has been seen more recently in specific health-intervention trials, for example smoking amongst high-risk or under-served groups [8]. Robinson et al [6] suggest that relatively little is known about effective retention methods due to a paucity of studies, though these do seem to be emerging [9–11]. It is clear that in some research areas, retention is a predominant concern and can require intensive effort and significant cost; for example participant retention in alcohol and other drug studies [12]. Meneses et al [13] report a retention figure of 77% of participants in a rural breast-cancer survivor’s study (over 12 months of the study) predominantly losing participants in the early stages of the work. In Acosta et al’s (2005) [14] study of a pesticide risk reduction programme 34% of migrant-farmworker participants were lost between a pretest and post-test period (227 vs 152). In Farquhar et al’s (2013) [15] study of Latino farmworkers: which included a variety of biomonitoring elements, attrition rates varied from 20.9% in year 1 (49 of 62 participants retained) of the study to 10.7% in year 2. In a study with farm owners and workers on prospective injury, retention rates were 85% and 86% over a 24-week period [16].

Considerable effort is required to reduce attrition using a range of retention strategies [11,13]. Regardless of the approach adopted, most studies are highly formalised with little (on-the-ground) flexibility and have a defined, research-office administered set of (retention) protocols determining, for example, the precise number of follow-up telephone calls [13] and associated letters to retain participants. Collins et al [8] indicate that ‘proactive + reactive’ approaches to recruitment to a second-hand smoke reduction trial study appeared to improve retention rates in contrast to ‘reactive’ ones: largely advertising based methods (94% compared with 74.7%). ‘Proactive’ approaches in this case involved the training of clinical staff to encourage patient participation directly. In this current investigation, we have adopted a different, more flexible, approach to ensure recruitment and continued involvement in biomonitoring based upon an emerging trend for more active involvement by members of the local population as researchers in the study.

## Communities Involved in Scientific Research

There is an established tradition that, particularly in scientific research seeking to inform future policy, direct involvement in the research process by communities can improve policy development [17,18]. This is particularly true for those which have a scientific context with potentially contentious impacts, notably where there are issues of environmental justice [19].

Morello-Frosch et al [20] showed the utility of community involvement in enhancing the rigour of science-based projects, when seeking to address community problems. Such direct participation in research takes various forms but is commonly defined as Community Based Participatory Research (CBPR) [18]. This direct involvement in research activity is also seen elsewhere, for example: in the generation of more active policy development [21,22]; for (environmental) learning [23] and in evaluation processes [24]. Collectively these various approaches have been drawn together as Public Participation in Scientific Research or PPSR [25].

Whilst not intending to be a fully-formed participatory project, our work does aim to adopt many of its underpinning principles by seeking to utilise the locally-based skills of community-recruited researchers to ensure quantitative rigour whilst acknowledging there are often complex ethical issues and challenges associated with such work [25,26].

We used community researchers to address directly the challenges presented by biomonitoring studies, particularly those associated with recruitment, and retention. The assumption in using community researchers was that they bring what Chisholm and Elden [27] refer to as the ‘tacit and explicit knowledge of insiders’ or more succinctly ‘cultural competence’ which other researchers may not possess [28]. The use of this more nuanced approach can avoid researchers making ‘cultural missteps’ [29]. The degree of embeddedness individual community researchers have in their respective communities will vary, but it is likely to be greater than that offered by ‘outsiders’. This is in line with Garnett et al’s [28] observation where their own core research team ‘lacked direct cultural involvement in the environments being studied’ and is thus comparable to the situation reported here.

In much of the CBPR work we see reference to this ‘insider’ perspective [30]; as researchers we need to be clear to what the ‘inside’ refers. In previous refugee-policy work community researchers were recruited on the basis of having a common experience of ‘flight’ [31]. For the purposes of the work presented here, we saw the common element, based upon an understanding of the geographic area and more importantly that community researchers had an awareness of the issues faced by farming and their surrounding communities.

In the work reported here, community researchers were recruited to ensure the methodological rigour of the study. By recruiting researchers from within the local geographic communities under study it was anticipated that enlisting farmer and resident participants would be more successful and that there would also be lower attrition rates. A community-researcher approach was deployed as their embeddedness within the case-study communities was likely to bring a range of community-based strengths and resources in order to:

- Aid the more effective recruitment and retention of participants, both farmers and nearby residents.
- Encourage participants to maintain their interest through the establishing of trusting relationships–and hence reduce attrition.
- Represent a continuing locally-based presence for the research project over its lifetime.
- Enable more effective responsiveness to emerging local issues and changing circumstances as the project progressed.
- Provide cultural competence in a local area, including understanding any local sensitivities; pesticide use is known to be an emotive subject in some communities [32,33].

The central concern in the proposed study was the collection of substantive data from samples of farmers and local residents (including children) over spraying seasons; in some localities over a period of 7 months in each of two consecutive years. Perceived inconvenience and commitment to provide repeated urine samples and complete the accompanying diary might be a deterrent to continued participation amongst residents; given the need to offer a regular commitment over several months (in most cases).

Overall, then the aim of this paper is to explore the potential in the use of community researchers as an effective means of recruiting participants and ensuring their continued involvement in a biomonitoring study. In addition it seeks to examine the challenges faced in a project which had a major time-sensitive element, specifically collecting key first-morning void urine samples within 48 hours of a pesticide spray event.

## Methodology

### Ethical Approval

The study has received full ethical approval by the NHS South East Scotland Research Ethics Committee (SESREC) 3 (study number 10/S1103/63) and participants provided their informed written consent to participate. Guardians of children provided written informed consent on the child’s behalf. Specific ethical approval was obtained from the School of Engineering and the Built Environment, Glasgow Caledonian University (GCU) for the GCU-led interviews and discussions with community researchers. The community researchers provided their informed written consent to participate in the interviews and discussions.

### Recruitment of community researchers

Over the whole project fieldwork, six community researchers were recruited through job advertisements emphasising the professional nature of the job. A clear specification was set out in the advertisement that the project sought to recruit those who had demonstrable knowledge of a local study area (i.e. cultural competence); an ability to engage actively and positively with the general public and ideally had knowledge of the farming community. Job advertisements were placed in local press and job centres in the geographical areas of interest (East Lothian, Kent and Norfolk). These three geographical areas, predetermined before community-researcher recruitment: East Lothian and Norfolk are major arable crop growing areas, whilst most of the orchards in the UK are located in South East England. Each of the geographical areas were large therefore researchers worked in sub-regional areas, which they helped to define, near their own locality: typically no more than a 1-hour driving distance radius from their home.

At interview, applicants were asked to demonstrate their social and geographic knowledge of the area and their ‘connectivity’ with parts of the farming community; revealing both their cultural awareness and ability to access relevant communities. This was necessary as the study, in individual sub-regional areas of considerable geographical scale, was not restricted to small, tightly-defined individual communities as is often the case in CBPR approaches for example. In that context, it was unreasonable to expect community researchers to have in-depth knowledge beyond their immediate own locality.

### Training

**Fig 1:**
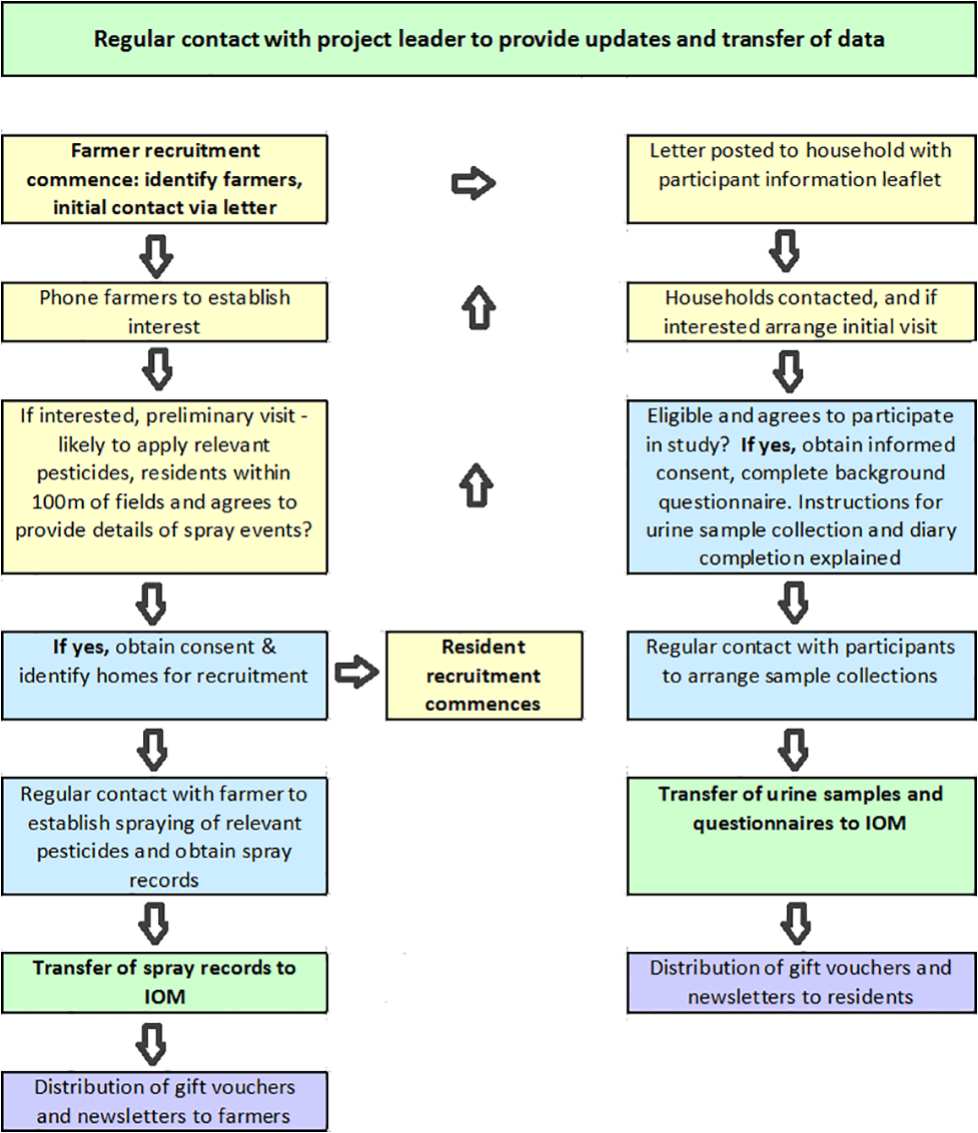
Process flows indicating roles of the community researchers.

The project leader gave each community researcher comprehensive training in their roles and responsibilities, the importance of complying with the study protocol (which itself set out the expected responsibilities of community researchers and their role in the project and which received ethical approval before their recruitment) [34]; how to identify and engage effectively with proposed study participants and, how to build and maintain these relationships for example by ensuring regular but not burdensome contact. Training involved drawing upon their own significant contribution to this process, based upon their considerable but varied local knowledge. In addition, the sessions considered health and safety (particularly ‘lone working’) arrangements, project-specific ethical issues and practical, mutually-agreed flexible working arrangements. For example, some researchers preferred direct employment whilst others preferred to have self-employment status. Fig 1 summarises the formal roles and responsibilities that the community researchers were expected to perform.

### Supporting community researchers

In the first stages of the study, on-site training support was provided, for example the project leader accompanied community researchers to early-stage interviews with farmers and residents. This provided both direct support and ensured the formal processes of the project were adhered to. Remote working by community researchers, both from one another and the core research team, meant fostering and maintaining good communication was essential to reduce any feelings of isolation and ensure that the study protocol [34] was followed. To help create and maintain a project-team community, weekly teleconferences were held between all the community researchers and the project leader. The latter also provided considerable individual support throughout their project involvement. Teleconferencing facilities also enabled exclusive discussion for community researchers only. Researchers were encouraged to act as ‘buddies’ to each other and to work out their own arrangements (usually in pairs) for work or locality-sharing, when working in the same region. Community researchers in each case study area, in consultation and occasional facilitation with the project leader, were encouraged to come to mutually-agreed arrangements for the apportionment of workloads to account for time capacity and travel distances when engaging both participant farmers and residents. Ultimately, an average of around 12 hours per week was spent on the project by each community researcher over their period of employment and occasionally travelling up to 300 miles in a week to collect samples and engage with participants. Additionally periodic project leader visits to each area were undertaken to discuss issues and to conduct auditing.

Whole-day debriefing sessions were held at the project headquarters at the end of each year’s sampling periods. The aim of these sessions was primarily for community researchers to share thoughts on emerging trends and issues in the research, to provide direct input into the research process, to help refine the developing strategy and to reflect upon their own experiences of the project. These debriefing sessions included individual interviews with each of the community researchers as well as group discussion sessions. Their observations then influenced specific new developments in the project’s management and of its research design notably impacting upon pesticide selection, as farmers informed community researchers directly about current, on-the-ground practice.

Consent was obtained from the community researchers to record both these individual and group debriefing sessions. During the interviews the community researchers were asked to reflect on their role and contribution to the project (consistent with Burns and Schubotz [35]). Interviews were undertaken by two researchers: one from within the lead organisation but having no involvement in the project (other than to conduct these interviews) and one from outside the lead organisation, involved in the project, but who had no involvement with the project’s day-to-day operation [PT]. The lead researcher [KG] had neither sight of the transcriptions nor heard the original interview audio recordings as she had a key role in providing support and supervision for the community researchers. In the results / discussion section we present community researchers’ reflections in line with McQuiston et al’s [36] adaptation of Fals Borda’s [37] two-language approach, in which we present their anonymised and essentially lay contributions.

## Research Process

### Farmer recruitment

The first and crucial stage in the process was the identification and recruitment, by community researchers, of suitable farms / farmers into the study: specifically those willing to provide details of their spray records, application of relevant pesticides and having residential areas adjacent to the fields likely to receive application of these pesticides. Without the recruitment of suitable farmers into the study, the other steps in Fig 1 would not follow.

Having identified possible farms (initially) using Ordnance Survey maps, phone and industry directories and their own local knowledge of each area, community researchers, with core research staff identified potential farms, farmers, and residents. Community researchers approached farmers by letter and then sought to meet them directly to provide further project details and discuss their willingness to participate and from initial contact to consenting to participate normally took 1 to 2 weeks. The process of recruitment took place in the late winter and spring of each of the study years to avoid the busiest times of the farming year.

### Resident recruitment

Having gained acceptance from the farmer responsible for spraying neighbouring fields–community researchers sought to recruit nearby residents (specifically those living within 100 metres of fields likely to be sprayed with ‘pesticides of interest’) and maintain their involvement over the period of a given season. The season can last for several months with spraying taking place intermittently over different periods within a calendar year. The project needed to engage and recruit adult participants (over 18 years) and through them, any children (aged 4–12 years) they were responsible for, living at the same residential unit. The key data-collection element of the project was to provide first morning void urine samples and complete a short activity questionnaire once a week on the same specified day for each participant and also reactively one day and two days following relevant spray events; where known. Hence, whilst the expectations were small in terms of the time taken, there was a need for participants to be consistent and reasonably methodical. At the same time the research team was aware that some might see the requests made of potential participants as unnecessarily intrusive. Participants could be reassured (and were specifically informed) that the study had received ethical approval from the NHS Research Ethics Committee. Allaying these possible concerns was a significant part of community researchers’ roles.

Residents were initially contacted by letter along with an information leaflet, to inform them of the project’s details. Residents were invited to return an acceptance slip if they were willing to participate. Community researchers would then follow this up by telephone to see if residents were interested and arrange a private visit to their home to describe more fully: the project and its aims; issues of anonymity and confidentiality as well as ethical issues associated with findings and the research process, and to emphasise this was not a ‘health study’. This latter point was of particular concern as, given the emotive nature of the use of pesticides in some areas [1–4]—it was essential that the terms of the study were clearly set out to allay any potential fears and also, to manage expectations. For example, participants were advised both in face-to-face conversations and in the project’s information leaflet that this was not a health study and that the project team would provide a summary of the urine sample results for all the samples analysed once the work was completed.

Community researchers then with willing participants established an individual protocol with each household for the collection of urine samples. Residents received £5 gift vouchers for each sample provided for the inconvenience it represented: a value consistent with other research [38], with payment for children’s samples being paid only directly to the responsible adult / parent at the same address. The appropriate value of gift vouchers was administered to participants at the end of each year of sample collection.

A common strategy adopted in a range of health-intervention studies is the use of ‘incentives’ for example [39] to reduce levels of attrition [7]. There is some more recent evidence that, in population-based cohort studies, incentives impact retention and that this improves with amount paid [11]. In our study these £5 payments were not ‘incentives’ but rather ‘compensation’ for inconvenience and intrusion as described in the study’s ethically-approved protocol.

## Results

There were three central elements to the project’s effectiveness, namely the recruitment of farms / farmers; recruitment of residents living within the designated distance and ultimately, the generation of sufficient, relevant, urine samples for laboratory and statistical analysis. Table 1 provides a summary of the proposed and actual overall recruitment of farms and residents into the study, with recruitment in both instances substantially exceeding expectations. The target numbers of residents for inclusion in the study was based on a range of conservative power calculations carried out for a number of pesticides identified as being most likely to be applied during the spray seasons in the target areas [34].

**Table 1:** Target and actual farmer and resident recruitment in 2011 and 2012.

	2011		2012	
	Target	Actual	Target	Actual
Farms	10	14	16	19
Residents	75	139	120	195

In addition a total of 3,275 urine samples were obtained over the sample collection period.

It emerged in the early stages of the project design that simply identifying farms using secondary source material on maps and from listings was a crude, and often misleading, form of identification; producing considerable inaccuracies. It was neither reliable as a way of identifying farmers growing appropriate crops and spraying pesticides / active ingredients of interest to the study, nor could it reveal the complex land-use and ownership patterns which were essential to ensure the correct farmers were approached: hence on-the-ground recruitment work using community researchers’ existing and accruing knowledge proved to be essential. As one community researcher noted:

“… finding out who owns those fields can be exceedingly difficult ….”

“It is a huge agricultural area with a low population density. It turned out to be a bit of a waste of time looking at Google maps because it would often turn out that the farm that was closest to the small towns were not actually owned by the farmer.”

Direct community-researcher interaction with farmers (or those subletting) emerged quickly as a central element to the research method enabling them to establish detail about the complexity of farming patterns, where, for example, farmers would rent out fields to other farmers if they weren’t needed during a particular season. This direct interaction was often grounded on locally-sourced contact information and, given the competence community researchers brought to the project, often based on their own knowledge and experience, for example of local farming practices.

## Practicalities

### Recruiting participant farmers

The mechanisms for establishing the contacts were seen as particularly important with email and phone as an initial contact approach seen as particularly unproductive as *“it was easy for you to be pushed off”*. Hence the direct physical interaction with farmers in order to establish, from the outset, an effective participatory relationship was seen as essential.

“Until you actually go to see them and the farm physically, you don’t really know whether or not they are going to be a good candidate for this [study].”

As a result of this learning and the strategies adopted, the community researchers were able to recruit farmers in excess of what was initially anticipated (Fig 1). There was strong evidence that this was because of the on-the-ground location and cultural competence which community researchers were able to bring to the project, for example through ‘snowballing’:

“… it helped… being a farmer’s daughter in the area, so I have a lot of family and friends that I spoke to and we got a good number of farms that we could then contact.”

“…there was a connection there… when most of the farmers met they said are you Y’s daughter… that helped the trust definitely and… that I knew … about farming…”

This competence included not only local connections but also an understanding of workloads, seasonal commitments and appropriate ways to approach individuals in this industry including what potential participant’s concerns might be. ‘Trust’ was particularly identified by one community researcher as the most important element for effective engagement.

“…I knew they would be in probably early in the morning for coffee time, [and] lunch time. Winter time is the best time to get anybody because they tend to be quieter. And I knew then don’t touch them when its harvest time as it gets busy and they are not wanting extra things to do really.”

“I realised that with spraying there is no particular pattern, ‘yes you like to do it on such and such [a date] but there is never a particular date in the diary and it is very weather dependent, so it’s a moveable feast’ …: I had that understanding already there, which helped I think.”

Nevertheless some farmers did express concerns and demonstrated an unwillingness or reluctance to participate, because of the potential for straining relationships with local residents and communities. Community researchers consistently reported that this first stage, the identification and recruitment of farmers, was the most difficult element of the research process.

“…for some farmers it was an issue as they didn’t really want to get members of the public perhaps worried about what was going on in the fields…”

“I think they were suspicious. Some of them I spoke to felt that they would be happy to be involved but they wanted to be good neighbours for their residents. They thought that by doing this and being involved in the project, it would make the residents suspicious and that would impact on their relationship with them. They didn’t want to stir up any trouble. Which is what a couple of them actually said “we don’t want bad feeling stirred up”.”

There also remained apparent suspicion elsewhere in the industry and a degree of peer pressure:

“… they [farmers] are speaking to each other and I think the ones that are taking part are going out on a limb… X had attended… a meeting the day before and he said to me everybody told me not to take part. He said they said don’t take part but I said it was up to me and I don’t think there is a problem.”

“One of the farmers did query the list of [pesticides] what we were looking at and asked if we were checking up on them (some on the list they are not allowed to use anymore). I reassured him it was just what we [could] test for and [we are] not trying to catch you [out] with anything.”

There were also some concerns expressed about the impact upon their business:

“I think the farmers feel that something might be taken away from them if the results were to show that a chemical, they really feel they need, creates problems for residents living nearby.”

Or that they might be losing part of their “arsenal” of pesticide “tools”.

There were then broader challenges which community researchers had to contend with and understand, notably the structural elements of farming practice which to some extent are generic, but others were locally specific.

### Relationships between farmers and residents

A particular concern amongst farmers when trying to recruit them as participants was their ongoing relationships with local people and communities:

“They [farmers] would say “we don’t want you knocking on our neighbour’s doors getting the residents scared of something that they are not currently afraid of”. Or we have only just put them at ease and you are going to come along and ruin that.”

Another noted farmers’ feelings:

“Some… had particular residents that would come out all the time when they started spraying and asking them what they were doing, saying ‘why couldn’t you wait until the wind is in a different direction?’ and another saying ‘there’s that horrible man coming again trying to kill us’: well that’s what he felt the residents were thinking.”

Clearly farmers were concerned:

“… to keep a good relationship with the [local] population and didn’t want anything to worry them.”

### Recruitment: Resident participants

There were a total of 296 participants recruited to the study (238 adults, 38 children). Of these just over half (149) provided at least one sample result that was related to a relevant spray event. The ultimate measure of the project’s ‘success’ was the ability to obtain a sufficient number of urine samples from the participants. A total of 3,275 urine samples were obtained, 1,587 of which were provided by eligible participants: defined as those who provided at least one, spray-event-related urine sample.

Whilst, amongst residents there was some ‘*hesitation’*, associated with practicality issues largely associated with the time commitments required, there was a recognition residents’ perspectives and associated concerns were different to those identified by farmers:

“…I think maintaining them [in the study] is a different kind of relationship. They are definitely coming into it from a different point of view. Some of them have various concerns about spraying…”

Community researchers noted that residents were often reassured when they were aware that local farmers were taking part in the study:

“Once we had identified the farms, recruitment of the residents was pretty easy and relatively straightforward because most of the people living in those areas do have concerns about pesticides and were more than happy to participate.”

There was also strong evidence amongst residents (as there was amongst farmers) of a philanthropic attitude towards involvement in the study with community researchers identifying that residents perceived a *‘greater good’* coming from ‘*a worthwhile project’* and their involvement which might in turn result in *‘actually helping people find out things…’*.

“…I think a lot of people are interested in what goes on and interested in taking part in a project that will potentially help people that live in the countryside in the long term.”

“Some people even said they don’t need the money for it, they were just glad to be involved in the project.”

The indications were that the two participant groups came to the project with differing perspectives (and possibly expectations), but as community researchers engaged over time with them, these coalesced as researchers indicated both participant groups sought reassurance but could also build upon considerable goodwill.

### Building relationships and cultural competence

Community researchers, whilst initially bringing a high degree of competence to the project also acknowledged that this further developed as the fieldwork progressed, particularly in the enhanced relationships with residents and farmers which ‘*got better*’ ‘*as time went on*’.

“…the length of the project is good because if it had only been, say one year, I would have firstly not developed such a good relationship with them and I really feel that is very much in place now. And that it’s taken quite a long time to get it in place.”

In addition community researchers were able to bring this competence into the project management. One, for example, provided text, based upon her knowledge of the area, for the locality-specific element of a project newsletter. This, as an engagement tool in itself, further enhanced her relationship with resident participants:

“…we did our newsletter and handed it to everybody at the end of the year, and one of our residents said to me whoever wrote our bit of the newsletter did it well … it was me so I was so pleased. Because basically I just explained what was going on in the fields…”

“… I am not saying that’s kept them along in the project but I think having a newsletter, … has kept people involved in where are they in this [project] … I have asked a few people did they read the newsletter and they think it’s great that we are doing it [elsewhere], so I think they feel involved in something that is country-wide now as well.”

The importance of establishing and maintaining the relationship was clearly identified:

“…having the one researcher dealing with them you get to understand their feelings and when they are beginning to change, so you know how to deal with it then… keeping that relationship going is quite important.”

### Retention

Though retention had been expected to be a particular challenge it proved not to be a significant issue on the project. This appears to have been as a direct result of the trust that community researchers were able to establish with farmers and residents. Meneses et al [13] and Cassidy et al [40] draw attention to the key role effective relationships between researchers and participants play in ensuring higher retention rates: the positive aspects of these proved to be fundamental to the effective administration and operation of this project. As a consequence only 26 participants (8.8% total resident participants) withdrew for reasons unconnected with the study process itself.

“Once we had the farmers… it was pretty easy to maintain their interest in it… in the end…farmers that we had were all really amenable.”

And again the philanthropy of farmer-participants emerges:

“…The farmers we have are very keen to do it as well because they don’t want to be using products that are harmful in any way and they want to see if there is exposure…”

Whilst retention of participants in the study was a concern initially there was also a need to release participants from the study both because of the seasonal variability in spraying patterns and the variations in pesticides used. The use of community researchers enabled the project to explain this without ‘discontinued’ participants feeling they had wasted their time.

## Data Collection Process

It proved highly effective having locally-based community researchers notably when being able to respond to specific spray events:

“…the farmer might contact us ‘I’ve just been in the field and I’ve done it [spraying]’, then you’re on the phone trying to let everybody [residents] know that I’m about to drop a [sampling] kit off to you for tomorrow morning. I think they grew to expect that that was okay and most people were pretty gracious about getting the call in the evening and then having somebody come out about 9.30 pm to drop off a kit. Once they got a sense of what was being asked of them it just got easier.”

Knowledge of the spray event either in advance or immediately after it had taken place and being able to respond to these was crucial to the collection of appropriate reactive samples. The major determinant in this was the relationship with the farmer and the ability to make timely contact with residents to ensure samples were collected within 24–48 hours. This responsiveness was a key element. Similarly good relationships were developed with farmer-participants so that they did not feel imposed upon.

“…one was very good in letting us know when, where and what active ingredients he was spraying and even let us know the weather conditions. I think he was interested in the science…”

“…just keeping it friendly, … for them they don’t want it too official (because they aren’t office people). I think you need to understand [that] to get their trust.”

This flexibility was also reflected in the nature and frequency of direct contact with farmers–for some there was a weekly interaction to view spray records, for others it was agreed between the community researcher and the farmer that this would be undertaken at the end of a spray season. Of course, the logistical elements did not always work:

“things that hasn’t worked so well… is the matching up with the spray events… due to them [farmers] planning to do it and then the weather changes or something else happens and then they just forget to call you back and let you know.”

In developing relationships with resident participants a similar high degree of flexibility was necessary: both in terms of logistics and direct participation. For example, there were some agreed sample-supply interruptions when residents were away or unable to provide them at a given time.

“…some of them were incredibly cooperative (let you know when they would be away, put their samples out) …”

Similarly communication methods were individually negotiated as the data-collection activity progressed:

“Some people just don’t want reminding they can do it perfectly well themselves and some wanted a text or email or something.”

Maintaining and developing professional relationships with participants needed tact and discretion:

“The majority leave it [the sample] outside. There is a few who like you [to] knock on the door and you have a wee chat, … you need to have the right balance of being chatty and not just rushing away. People tell me all sorts of things and I keep it all very professional but they still… want to chat.”

But similarly there were practicalities that community researchers identified as not working:

“The diary I think is slightly fiddly but we did review it. Nobody has complained about it as such but if you ask what was the most silly part about it they would probably say filling in the diary: having to think back 2 days.”

The indications were that once the relationships were established with participants; individual protocols agreed and patterns for sample collection bedded down the project progressed smoothly.

## Discussion

Whilst there are acknowledged concerns over the rigour associated with data collected by community researchers [28], there is a strong indication from our study that the use of a community-researcher methodology can enhance the rigour of the work as data quantity and its quality can be improved by it.

There are clear advantages to the use of a community-researcher approach for biomonitoring studies. They bring knowledge of the community under study; what is referred to as their ‘prior tacit knowledge’ [28] and over the period of the work, being on the ground, developed further a considerable level of cultural competence amongst (in this case) farming communities. This competence, built into the recruitment process for community researchers, was further enhanced during the research activity. Community researchers were able to bring a real depth of locally-specific knowledge enhancing the research design, recruitment process, project credibility and trust. This enabled, particularly in the first instance, effective recruitment of farmers, identified by community researchers as the most challenging part of the process. They were able to ensure that we approached potential participants in the most appropriate way, but also that as the community researchers developed trusting relationships the learning from this was carried forward into recruitment and engagement activities in subsequent research localities as they came on-stream. But to ensure all of this both substantial training and particularly ongoing support were essential; as also found with a different community-researcher demographic cohort [35]. This was necessary in our study for both operational reasons and also because of their remote working locations.

The enhancement of trust enabled effective relationships to be built both with farmers and residents, and ensured, in this case, large-scale sampling using a rigorous approach. It also enabled responsiveness to local circumstances affecting directly the research process: allowing a high degree of flexibility to, often, fluid farming practices—most notably with respect to spray events. By their nature these are unpredictable and at the same time were a major component in determining data quality. In addition, community researchers provided insights into farming-community dynamics and pre-existing community relationships. This approach particularly revealed, for example, suspicions, in some quarters, amongst both sets of participants. A lack of knowledge of what farmers do produced, in some residents, suspicion about the potential harm associated with spraying practices. Equally it revealed farmer’s own worries that this study (and other similar ones) might disrupt their relationships with residents and, for some, with their contemporaries in the industry and other stakeholders. The approach also revealed farmers’ anxieties about the impact the work might have upon their longer-term farming practice–notably what active ingredients they might be allowed to use, whilst at the same time many had concerns to make sure that they only used safe products.

There was then a direct impact upon the research process and practice produced by community researchers as they were able to articulate insight and knowledge of issues with respect to farming practices through this enhanced competence and hence ensuring the study protocol was rigorously followed Additionally they were able to inform appropriate research practice with respect to geographically-specific issues and also methodological ones, for example who to approach, where and when. The trusted status which community researchers attained made it also an effective way of enhancing communication playing a key role in the essential dissemination activities as a transparent part of the research.

Working with community researchers proved to be highly effective and ‘successful’ in facilitating the collection of over 3,000 urine samples which were used to answer the research aims and objectives. The approach is one however which requires considerable input by core research team members in ensuring the right support and supervision is provided, not simply with regard to community researchers’ own skill development but when necessary, to ensure effective quality control.

There are considerable challenges faced by those seeking to enlist participants into biomonitoring-exposure studies. The use of community researchers building upon their locally-developed cultural competence can provide considerable benefit to the effective management and success of such studies and can enhance its quality.
